# [Expression of Concern] Electroacupuncture at GV20-GB7 regulates mitophagy to protect against neurological deficits following intracerebral hemorrhage via inhibition of apoptosis

**DOI:** 10.3892/mmr.2026.13870

**Published:** 2026-04-01

**Authors:** Ruiqiao Guan, Zhihao Li, Xiaohong Dai, Wei Zou, Xueping Yu, Hao Liu, Qiuxin Chen, Wei Teng, Peng Liu, Xiaoying Liu, Shanshan Dong

Mol Med Rep 24: 492, 2021; DOI: 10.3892/mmr.2021.12131

Following the publication of this paper, it was drawn to the Editor's attention by a concerned reader that, in comparing the bar charts shown in Fig. 2D and F on p. 5 representing the quantification of the western blot data featured in Fig. 2C and E respectively, these bar charts were strikingly similar, suggesting that the same chart may have erroneously been included in this figure twice to represent the different experimental conditions.

The authors have been contacted by the Editorial Office to offer an explanation for this apparent anomaly in the presentation of the data in this paper, and we are awaiting their response. Owing to the fact that the Editorial Office has been made aware of potential issues surrounding the scientific integrity of this paper, we are issuing an Expression of Concern to notify readers of these potential problems while the Editorial Office continues to investigate this matter further.

